# Too well to die; too ill to live: an update on the lifespan versus health span debate

**DOI:** 10.7189/jogh.15.03022

**Published:** 2025-05-12

**Authors:** Deepak Jugran

**Affiliations:** Department of Sociology, Panjab University, Chandigarh, India

## Abstract

Since the dawn of human civilisation, the pursuit of immortality has been a perennial quest. Over the past century, unprecedented advancements in medical science, public health initiatives, and social policies have significantly increased the global human lifespan. The debate between lifespan (the total number of years a person lives) and health span (the period of life free from chronic disease or disability) has gained considerable attention in both scientific and public discourse, with global focus now gradually shifting from merely living longer to living better. With this increase, a critical question has emerged: are these additional years truly spent in good health? As the global elderly population projected to reach 2.1 billion by 2050, this demographic shift is expected to place a substantial burden on health care systems, economic structures, and social frameworks. Emerging research in epigenetics, regenerative medicine, and lifestyle medicine suggests that preventive measures and personalised interventions can compress morbidity, promote healthier ageing trajectories, and ultimately increase health span. We review current lifespan and health span frameworks to foster dialogue among basic scientists, clinical specialists, social scientists, public health experts, and policymakers, advocating for a balanced approach that prioritises extending healthy, functional years of life over simply increasing lifespan.

Since antiquity, human beings have been driven by an enduring desire to achieve immortality. Over the last century, significant advances in medical science, public health measures, and social policies have collectively contributed to a remarkable increase in global lifespan, transforming once-fatal conditions into manageable ones and drastically reducing mortality [1]. However, this increase raises a significant concern: are these additional years of life also spent in good health? The ongoing discourse surrounding lifespan and health span has emerged at a critical intersection of clinical and social sciences, emphasising the necessity for interdisciplinary collaboration in devising comprehensive responses to this multifaceted challenge. The focus is gradually shifting away from merely prolonging life toward enhancing the quality of life during the extended years [2].

The global population aged ≥60 years reached one billion in 2020, constituting 13.5% of the world’s 7.8 billion – a two-and-a-half-fold increase from 1980. Projections suggest that this figure will rise to nearly 2.1 billion by 2050 [3]. This is concerning, as we are fundamentally unprepared for a demographic shift of this magnitude at the individual, societal, and governmental levels. As people live longer, they increasingly face challenges such as chronic health issues, social isolation, and uncertainty about the process of dying. This situation necessitates a shift in focus from merely extending the lifespan to improving the health span. In this viewpoint, we explore the scientific, ethical, economic, and policy considerations surrounding this debate. Critically evaluating these perspectives offers valuable insights into how medical, societal, and governmental priorities can be shaped to optimise longevity and overall well-being. Ultimately, the question remains: should we merely add years to life, or should we also enhance the quality of those extra years? The ongoing debate calls for an empirical and balanced approach that prioritises extending healthy, functional years of life.

## CURRENT INSIGHTS AND UNCERTAINTIES

The social and economic implications of increasing the human lifespan without a corresponding improvement in health span are significant. Prolonging life without improving its quality places a significant burden on healthcare systems, reduces productivity, and diminishes the overall well-being of both individuals and society [4]. A recent McKinsey report estimated that the global economic cost of ill health was nearly USD 12 trillion in 2017, which was 15% of the global gross domestic product at the time [3]. Over the past twenty years, the combined costs of healthcare expenditures, lost productivity, and reduced labour force participation due to illness have resulted in a staggering financial loss of USD 47 trillion worldwide [4]. With the global elderly population expected to reach 2.1 billion by 2050, there is an urgent need for a well-defined framework that integrates lifespan, health span, and economic sustainability [3]. Such a framework is essential to manage the rising burden of chronic diseases, reduce healthcare costs, and ensure that ageing populations can lead healthy, independent, and economically productive lives. 

From a scientific perspective, several factors influence lifespan and health span, like genetics, lifestyle choices, environmental exposures, and access to healthcare. Recent advances in geroscience, regenerative medicine and lifestyle medicine suggest that we can potentially delay the onset of chronic diseases and promote healthier ageing [5,6]. In recent years, the emerging field of epigenetics has facilitated numerous studies exploring how environmental and behavioural factors influence gene regulation and their subsequent contributions to overall quality of life [7]. While there is no consensus on how to balance the trade-offs between lifespan and health span, given the complexity of the biological, environmental, and behavioural pathways involved, a balanced approach could hold the key to a healthier, more functional ageing process. Nevertheless, some researchers argue that biomedical advancements targeting lifespan extension may simultaneously promote health span, as delaying the ageing process could defer the onset of chronic diseases [8]. In contrast, others contend that medical interventions focussed solely on prolonging life might lead to longer period of suffering and disability, ultimately reducing overall quality of life [9].

Current medical interventions in chronic conditions often extend life, but do not fully cure the underlying diseases. They generally focus on managing symptoms or slowing disease progression rather than completely restoring functional capacity, leading to prolonged periods of frailty, disability, or dependence [4,10]. In recent years, non-communicable diseases such as cardiovascular diseases, cancers, chronic respiratory diseases, and diabetes have become the leading causes of mortality globally, accounting for nearly 40 million or 71% of all deaths annually [4]. Similar trends have also been reported in India, where chronic diseases are on the rise and have become a major cause of mortality, even in rural areas [11].

Geographical and cultural differences significantly shape how societies approach ageing and chronic diseases, directly impacting the balance between lifespan and health span. In developed countries, a focus on technological innovation and preventive care often extends both lifespan and the period of healthy living [12]. Conversely, in many developing regions like sub-Saharan Africa and parts of Asia, reliance on traditional practices and constrained healthcare resources may extend lifespan modestly but often without a parallel improvement in health span, thereby widening the global disparity in ageing outcomes [12].

## LIFESPAN: THE CONUNDRUM OF LIFE EXTENSION

The increase in lifespan is indeed one of the most celebrated human achievements of the past century. Just a century ago, global life expectancy averaged around merely 35 years; today, it stands at 72 years [13]. This unprecedented rise in the human lifespan, fuelled by advancements in medicine, sanitation, and nutrition, represents a triumph of modern medicine. Nevertheless, this achievement is increasingly overshadowed by a paradox; while humans live longer than ever, they are not necessarily healthier.

The surge in chronic diseases not only imposes a heavy economic and psychological burden on individuals and society, but further exacerbates social inequalities. The ethical and existential dilemmas surrounding the extension of the lifespan are equally challenging and complex. As a result of growing social inequalities, there are fears that it may create a two-tiered longevity system within the existing social structure, where those with higher incomes enjoy longer and healthier lives due to better access to preventive healthcare, advanced medical treatments, and supportive lifestyles. Meanwhile, disadvantaged populations are more likely to face prolonged periods of poor health and limited access to interventions that enhance health span [14]. Furthermore, bioethicists also argue that indefinite extension of the human lifespan disrupts the natural cycle of existence and generational turnover, raising concerns about planetary sustainability [15]. Advances in CRISPR gene editing, senolytic drugs, and regenerative therapies fuel speculation about a future in which ageing will be treated as a disease [16]. Countries such as Japan, Italy, and Germany, with individuals aged ≥65 years exceeding 20% of their population, already face challenges such as rising healthcare expenditures, pension crises, and increasing dependency ratios [17].

The increase in longevity also raises important questions about the process of dying. A comprehensive report by the Institute of Medicine Committee on Care at the End of Life in the USA suggests that, while death itself may not be a significant societal concern, the quality of the process of dying is critically important [12]. The report further suggests that modern medical technology has, at times, overshadowed compassionate care, leading to scenarios where patients experience prolonged suffering at the end of their lives. Moreover, an increase in longevity may also strain the economic and social systems; hence, the authors of the report recommended institutional measures to address the following four major deficiencies in the current medical care.

Firstly, too many individuals suffer needlessly due to errors of omission, where caregivers fail to provide adequate care, and errors of commission, where ineffective or even harmful treatments are administered. Secondly, legal, organisational, and economic barriers obstruct the delivery of high-quality end-of-life care. Outdated and scientifically flawed drug-prescribing laws, restrictive regulations, and rigid interpretations by state medical boards further complicate patient management. Thirdly, the education and training of physicians and healthcare professionals remain inadequate, failing to equip them with the necessary attitudes, knowledge, and skills to provide compassionate and effective care to dying patients. Lastly, current knowledge and research are insufficient to guide evidence-based studies on the dying process.

Biomedical and clinical research has predominantly focused on disease detection, treatment, and prolonging life, often neglecting the complexities of end-of-life care and the quality of the dying process. As Sir William Osler famously stated, we must ‘care always’ – a principle that gains renewed significance when the possibility of ensuring a healthy lifespan diminishes. In such instances, it becomes imperative to approach the dying process with dignity, compassion, and a commitment to alleviating neglect and emotional suffering [12].

## HEALTH SPAN: INCREASING THE QUALITY OF EXTENDED YEARS

A healthy lifespan is a critical concern, given its substantial social and economic implications for both individuals and society. While its long-term benefits will also impact younger generations, older adults will experience the most immediate gains [18]. At the individual level, lifestyle modifications are the most effective tools for enhancing the health span, with research indicating that diet, physical activity, mental engagement, and sleep quality are key to these efforts [16]. These changes contribute to longevity and improve the quality of life in the extended years [3]. In addition, social, intellectual, and physical engagements are also crucial in promoting healthier and longer lives [18]. While individuals can take proactive measures, the environment heavily influences their success, and hence, communities must foster age-friendly infrastructure, social support networks, and workplace policies to accommodate the ageing population [19]. There is growing concern that medical technology, combined with unhealthy lifestyles, may lead to a prolonged and undignified ageing and dying process. Many individuals now fear both overtreatment and abandonment during the final stages of their lives [12].

Health span gained prominence as a critical indicator about 30 years ago due to evolving interpretations of population health trends [18]. To address this, future research must develop a comprehensive framework to empirically measure, improve, and increase the health span. The World Health Organization introduced Healthy Life Expectancy (HALE) as a key metric for assessing quality of life alongside life expectancy. It is now widely accepted as a measure of population health at both the global and national levels. Estimating HALE involves determining the average age of onset for chronic diseases with their incidence rates. Current estimates indicates that roughly 20% of a person’s life is spent in poor health [18].

The failure to improve the health span alongside lifespan overburdens healthcare systems, leads to social isolation, triggers mental health crises, and increases strain on caregivers [10]. This situation brings up ethical issues such as whether a longer lifespan also means enduring decades of suffering, dependency, and financial insecurity [19]. Thus, a shift from a disease-centric approach to a proactive, preventive healthcare model is crucial, requiring collective efforts from individuals, communities, and governments to ensure that longevity is accompanied by well-being [20]. Nevertheless, without equitable access to preventive health care practices, longevity interventions may favour the privileged, exacerbating health disparities across socioeconomic groups [14].

## CONCLUSIONS: BALANCING LIFESPAN AND HEALTH SPAN

The modern longevity revolution has given humanity the ability to extend lifespan, yet this achievement has led to unique challenges. Historically, two distinct perspectives have shaped the ongoing debate surrounding lifespan. The first, known as the ‘failure of success’ argument, posits that extending life in individuals with poor health could adversely affect the overall health of the population [21]. According to this view, prolonging the lives of unhealthy individuals might lead to an increased burden of disease, placing a significant burden on health care systems, families, and society at large. The second perspective, however, focusses not only on extending life expectancy, but also on delaying the onset of diseases. By compressing the period of illness and disability into the final stages of life, allowing individuals to experience longer lives with fewer years of poor health, thus enhancing overall well-being [4,21]. In the near future, Artificial intelligence can play a significant role in extending health span by enabling early diagnosis, personalised treatment, and customised lifestyle interventions [22]. By analysing extensive healthcare data sets, algorithms can identify risk factors and optimise care strategies, ultimately extending healthy years.

The emerging discipline of lifestyle medicine has been recognised for its potential to optimise ageing trajectories and enhance well-being by extending health span [23]. It focusses on preventive measures to delay or reduce morbidity, physical and cognitive decline, and the onset of frailty, and is an evidence-based approach that helps individuals and families adopt healthy behaviours that significantly impact their health and quality of life [24]. In 2015, the Board of Lifestyle Medicine outlined essential competencies for managing key lifestyle factors, including tobacco use, alcohol consumption, diet, physical activity, weight, stress, sleep, and emotional well-being [25]. There is an urgent need to establish similar departments in all universities and medical schools worldwide to conduct evidence-based research on lifestyle choices individuals make during different phases of their lives. This research could then guide community and policymakers in effectively allocating resources to promote the healthy lifestyles necessary for extending the health span of individuals. It aligns with the most well-known framework for describing successful ageing by Rowe and Kahn in 1998, where they included the following three components for a disease-free, healthy ageing [23]:

1. Avoiding disease.
2. Maintaining cognitive and physical function.
3. Preserving engagement.

Although much work has yet to be done to fully understand the connections between lifestyle and healthy ageing, there is a growing body of literature to suggest that these connections exist as several of these lifestyles (nutrition, physical activity, stress) and geriatric domains (multimorbidity, cognition, physical function, engagement, frailty) are closely linked [23]. Lester Breslow was one of the first investigators to examine the connections between lifestyle and health in 7000 subjects, successfully demonstrating that individuals with a healthy lifestyle experienced an increase in longevity and a compression of morbidity [23].

Governments worldwide need to adopt policies that prioritise preventive care, investing in ageing research, and ensuring equitable access to health care and long-term wellness programmes [26]. In the absence of systemic measures, the burden of ageing will continue to fall disproportionately on vulnerable populations, exacerbating existing socioeconomic inequalities [14]. Public awareness campaigns ought to inform people about the importance of lifestyle choices, mental resilience, and community engagement in ageing well. By synchronising policies to extend both lifespan and health span, society can promote a more equitable and sustainable future – one in which additional years of life are marked by vitality, independence, and a high quality of life.
